# Updated rapid risk assessment from ECDC on the novel coronavirus disease 2019 (COVID-19) pandemic: increased transmission in the EU/EEA and the UK

**DOI:** 10.2807/1560-7917.ES.2020.25.10.2003121

**Published:** 2020-03-12

**Authors:** Eurosurveillance editorial team

**Affiliations:** 1 European Centre for Disease Prevention and Control (ECDC), Stockholm, Sweden

The European Centre for Disease Prevention and Control (ECDC) provides regularly updated information on coronavirus disease-2019 (COVID-19) relevant to Europe on a dedicated webpage. Besides general information including Q&As, daily case counts, and maps with disease distribution, examples of latest updates comprise: Poster: Effective hand-washing, COVID-19 - How to minimise the spread? and Considerations relating to social distancing measures in response to the COVID-19 epidemic. ECDC also publishes regular risk assessments and the Box below contains the summary from the sixth update published on 12 March 2020.

Box **Summary of the ECDC rapid risk assessment from 12 March 2020**

On 31 December 2019, a cluster of pneumonia cases of unknown aetiology was reported in Wuhan, Hubei Province, China. On 9 January 2020, China CDC reported a novel coronavirus as the causative agent of this outbreak, which is phylogenetically in the SARS-CoV clade. The disease associated with the virus is referred to as novel coronavirus disease 2019 (COVID-19).

As of 11 March 2020, 118,598 cases of COVID-19 were reported worldwide by more than 100 countries. Since late February, the majority of cases reported are from outside China, with an increasing majority of these reported from EU/EEA countries and the UK.

The Director General of the World Health Organization declared COVID-19 a global pandemic on 11 March 2020.

All EU/EEA countries and the UK are affected, reporting a total of 17,413 cases as of 11 March. Seven hundred and eleven cases reported by EU/EEA countries and the UK have died. Italy represents 58% of the cases (n=10,149) and 88% of the fatalities (n=631). The current pace of the increase in cases in the EU/EEA and the UK mirrors trends seen in China in January-early February and trends seen in Italy in mid-February.

In the current situation where COVID-19 is rapidly spreading worldwide and the number of cases in Europe is rising with increasing pace in several affected areas, there is a need for immediate targeted action. The speed with which COVID-19 can cause nationally incapacitating epidemics once transmission within the community is established, indicates that in a few weeks or even days, it is likely that similar situations to those seen in China and Italy may be seen in other EU/EEA countries or the UK.

There are no vaccines available and there is little evidence on the effectiveness of potential therapeutic agents.

In addition, there is presumably no pre-existing immunity in the population against the new coronavirus and everyone in the population is assumed to be susceptible. Clinical presentations of COVID-19 range from no symptoms (asymptomatic) to severe pneumonia; severe disease can lead to death. While the majority of cases (80%) are milder respiratory infections and pneumonias, severe illness and death is more common among the elderly with other chronic underlying conditions, with these risk groups accounting for the majority of severe disease and fatalities to date.

The risk of severe disease associated with COVID-19 infection for people in the EU/EEA and UK is currently considered moderate for the general population and high for older adults and individuals with chronic underlying conditions, based on the probability of community transmission and the impact of the disease.

The risk of healthcare system capacity being exceeded in the EU/EEA and the UK in the coming weeks is considered high. The impact and risk assessment on health system capacity can be mediated by the application of effective infection prevention and control and surge capacity measures.

The risk of transmission of COVID-19 in health and social institutions with large vulnerable populations is considered high. The impact of transmission in health and social institutions can be mediated by the application of effective infection prevention and control and surge capacity.

The EU/EEA and the UK are quickly moving toward a scenario of sustained community transmission of COVID19. The situation is evolving very quickly and a rapid, proactive and comprehensive approach is essential in order to delay transmission, as containing transmission to local epidemics is no longer considered feasible. A rapid shift from a containment to a mitigation approach is required, as the rapid increase in cases, that is anticipated in the coming days to few weeks may not provide decision makers and hospitals enough time to realise, accept and adapt their response accordingly if not implemented ahead of time. Measures taken at this stage should ultimately aim at protecting the most vulnerable population groups from severe illness and fatal outcome by reducing transmission and reinforcing healthcare systems.

Given the current epidemiology and risk assessment, and the expected developments in the next days to few weeks, the following public health measures to mitigate the impact of the pandemic are necessary in EU/EEA countries:

- Social distancing measures should be implemented early in order to mitigate the impact of the epidemic and to delay the epidemic peak. This can interrupt human-to-human transmission chains, prevent further spread, reduce the intensity of the epidemic and slow down the increase in cases, while allowing healthcare systems to prepare and cope with an increased influx of patients.
  Such measures include:
    - the immediate isolation of symptomatic persons suspected or confirmed to be infected with COVID-19;
    - the suspension of mass gatherings, taking into consideration the size of the event, the density of participants and if the event is in a confined indoor environment;
    - social distancing measures at workplaces (for example teleworking, suspension of meetings, cancellation of non-essential travel);
    - measures in and closure of schools, taking into consideration the uncertainty in the evidence of children in transmitting the disease, need for day care for children, impact on nursing staff, potential to increase transmission to vulnerable grandparents;
    - cordon sanitaire of residential areas with high levels of community transmission.
- Ensuring the public is aware of the seriousness of COVID-19. A high degree of population understanding, solidarity and discipline is required to apply strict personal hygiene, coughing etiquette, self-monitoring and social distancing measures. Community engagement and acceptance of stringent social distancing measures put in place are key in delaying and reducing further spread.
- Prevention and control of COVID-19 in hospitals and long-term care facilities is an immediate priority in order to: (1) slow the demand for specialised healthcare, such as ICU beds; (2) safeguard populations vulnerable to severe outcomes of infection (3); protect healthcare workers that provide care; (4) minimise the export of cases to other healthcare facilities and the community.
- Every healthcare facility should initiate training for all staff and those who may be required for healthcare provision during surge capacity. Countries should identify healthcare units that can be designated to care for COVID-19 cases, to minimise transmission to non-cases and to conserve PPE. Countries and healthcare institutions should identify additional facilities that can be used for the cohorting of cases with mild symptoms, in the event that surge capacity is exceeded by healthcare facilities. The highest priority for use of respirators (FFP2/3) are healthcare workers, in particular those performing aerosol-generating procedures, including swabbing.
- If resources or capacity are limited, rational approaches should be implemented to prioritise high-yield actions, which include: rational use of confirmatory testing, reducing contact tracing to focus only on high-yield contacts, rational use of PPE and hospitalisation and implementing rational criteria for deisolation. Testing approaches should prioritise vulnerable populations, protection of social and healthcare institutions, including staff.
- National surveillance systems should initially aim at rapidly detecting cases and assessing community transmission. As the epidemic progresses, surveillance should monitor the intensity, geographical spread and the impact of the epidemic on the population and healthcare systems and assess the effectiveness of measures in place. In circumstances with capacity shortages and strict implementation of social distancing measures, surveillance should focus on severe acute respiratory infections, sentinel surveillance in outpatient clinics or collection of data through telephone helplines.

A strategic approach based on early and rigorous application of these measures will help reduce the burden and pressure on the healthcare system, and in particular on hospitals, and will allow more time for the testing of therapeutics and vaccine development.

What is new in this update?

- Updated data on the epidemiological situation in the EU/EEA and the UK
- Recent findings on disease and transmissibility, including during the asymptomatic period
- Risk associated with COVID-19 for people from the EU/EEA and the UK
- Risk of local and widespread national community transmission in the EU/EEA and UK in the coming weeks
- Risk to healthcare systems capacity being exceeded in the EU/EEA and the UK in the coming weeks
- Risk of transmission of COVID-19 in health and social institutions with large vulnerable populations
- Options for preparedness and response focused on the mitigation phase, including rational measures in case of resource constraints or shortages
- Updated surveillance objectives and methods for the mitigation phase.

Source: European Centre for Disease Prevention and Control (ECDC). Rapid risk assessment: Novel coronavirus disease 2019 (COVID-19) pandemic: increased transmission in the EU/EEA and the UK – sixth update. ECDC: Stockholm; 12 March 2020. Available from: https://www.ecdc.europa.eu/sites/default/files/documents/RRA-sixth-update-Outbreak-of-novel-coronavirus-disease-2019-COVID-19.pdf
COVID: coronavirus disease; ECDC: European Centre for Disease Prevention and Control; EU/EEA: European Union/European Economic Area; ILI: influenza-like-illness; SARS-CoV: severe acute respiratory syndrome coronavirus; UK: United Kingdom.

